# Supplementary material for: Nirmatrelvir treatment of SARS‐CoV‐2‐infected mice blunts antiviral adaptive immune responses
Source: EMBO Mol Med. 2023 Mar 22;15(5):e17580. doi: 10.15252/emmm.202317580 (PMC10165354; doi:10.15252/emmm.202317580)
Supplement: Supplementary file 1 — Appendix [file EMMM-15-e17580-s003.pdf]

## Appendix

## Table of content

Appendix Figure S1 – S8

Appendix Table S1 – S2

**Appendix Figure S1.** ***Biochemical activity, antiviral efficacy, and plasma concentrations of nirmatrelvir.***

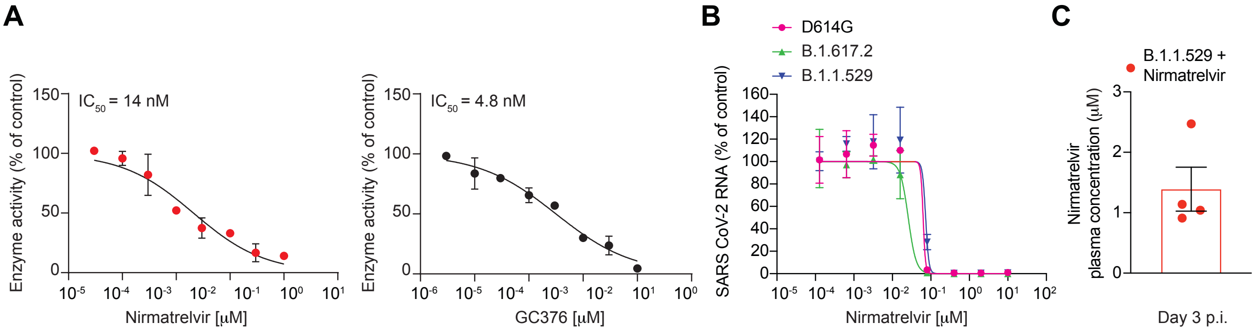


**(A)** Dose-dependent inhibition of nirmatrelvir (left panel) and GC376 (right panel) on SARS-CoV-2 $M^{pro}$. The enzymatic reaction was immediately initiated with the addition of the substrate. Data represent the mean ± SD of three biological replicates and are representative of three technical replicates.

**(B)** Dose-dependent antiviral activity of nirmatrelvir in HEK293T-hACE2 cells infected with SARS-CoV-2 D614G (purple symbols), B.1.617.2 (green symbols) and B.1.1.529 (blue symbols). Antiviral activity was determined by qPCR quantification of SARS-CoV-2 RNA in the supernatant. Data represent the mean ± SD of three biological replicates and are representative of three technical replicates.

**(C)** Nirmatrelvir concentration (µM) in the plasma of mice treated as described in **Figure 1D**. Data are represented as mean ± SEM and are representative of at least two independent experiments.

**Appendix Figure S2.** ***Absence of detectable SARS-CoV-2 specific T cells in the blood of K18-hACE2 transgenic mice infected with B.1.1.529*.**

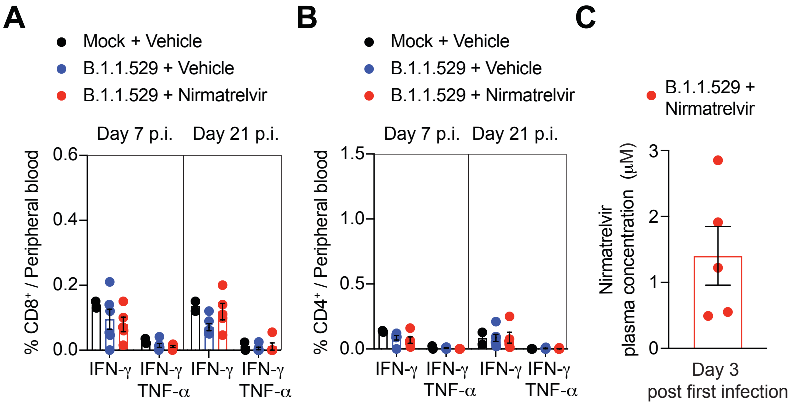


**(A, B)** Frequency of IFN-γ– and TNF-α–producing CD8$^+$ T cells (**A**) or CD4$^+$ T cells (**B**) in the peripheral blood of the indicated mice 7 and 21 days after the first infection ($n = 5$ or 6). Cells were stimulated *in vitro* with a pool of SARS-CoV-2 peptides for 4 hours at 37°C. Plots were pre-gated as (**A**) live$^+$/ B220$^-$/CD19$^-$/CD4$^-$/CD8$^+$ cells or (**B**) live$^+$/B220$^-$/CD19$^-$/CD8$^-$/CD4$^+$.

**(C)** Nirmatrelvir concentration (μM) in the plasma of mice treated as described in **Figure 2A**, 3 days after the first infection ($n = 5$).

Data information: Data are represented as mean ± SEM and are representative of at least two independent experiments.

**Appendix Figure S3.** ***Lymphocytic aggregates in the lungs of vehicle- but not nirmatrelvir-treated mice.***

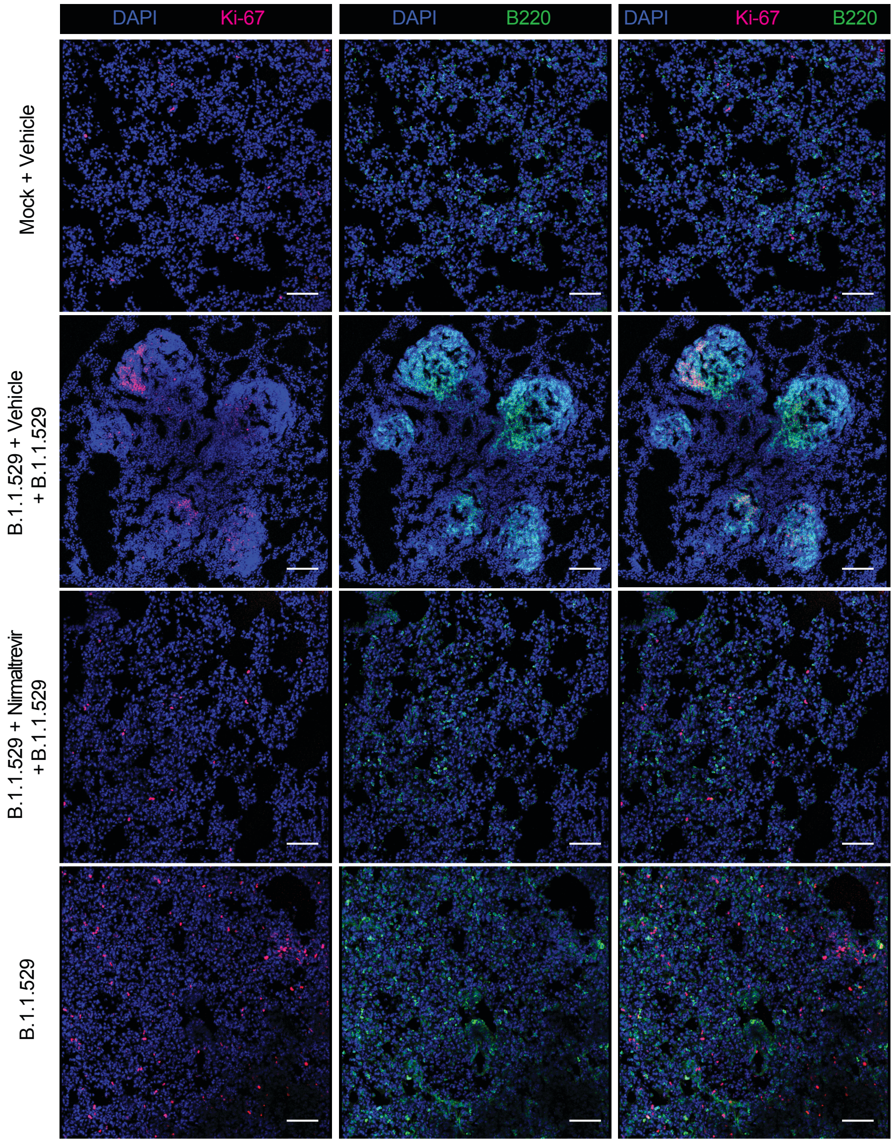


Representative confocal immunofluorescence micrographs of lung sections from mice 4 days post re-challenge, as described in **Figure 2A**. Mock-treated mice (first lane), vehicle-treated mice (second lane), nirmatrelvir-treated mice (third lane) and naïve-challenged mice (fourth lane). Ki-67$^+$ cells are depicted in purple; B220$^+$ cells are depicted in green and cell nuclei in blue. Scale bars, 50 µm.

**Appendix Figure S4.** ***Mouse-adapted SARS-CoV-2 replicates robustly in mice and maintains its sensitivity to nirmatrelvir.***

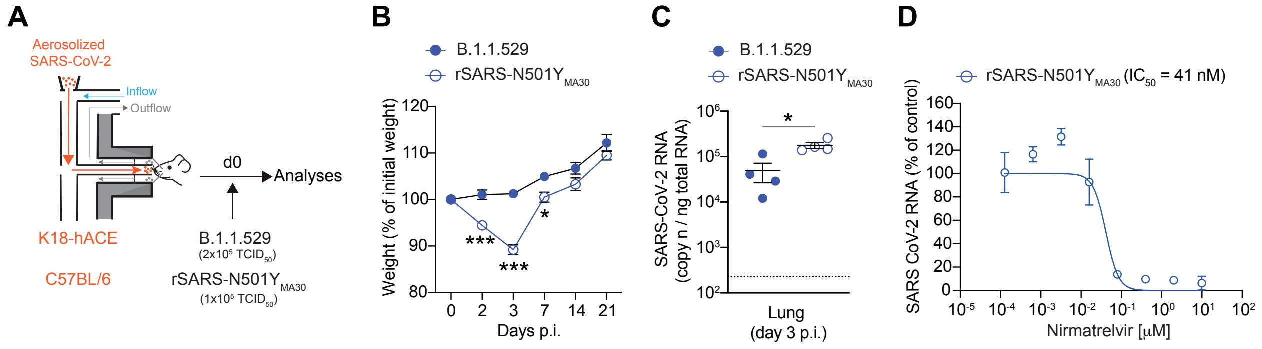


**(A)** Schematic representation of the experimental set up. Non-anesthetized K18-hACE2 mice (full blue dots, *n* = 4) or C57BL/6 mice (empty blue dots, *n* = 4) were exposed, respectively, to a target dose of 2 or 1 x $10^5$ $TCID_{50}$ of aerosolized SARS-CoV-2 B.1.1.529 or rSARS-N501Y$_{MA30}$.

**(B)** Mouse body weight was monitored daily and is expressed as percentage of weight relative to the initial weight.

**(C)** Quantification of SARS-CoV-2 RNA in the lung 3 days after infection. RNA values are expressed as copy number per ng of total RNA and the limit of detection is indicated as a dotted line.

**(D)** Dose-dependent antiviral activity of nirmatrelvir in HEK293T-hACE2 cells infected with rSARS-N501Y$_{MA30}$. Antiviral activity was determined by qPCR quantification of SARS-CoV-2 RNA in the supernatant.

Data information: Data in **B-C** are expressed as mean ± SEM; data in **D** represent the mean ± SD of three biological replicates and are representative of three technical replicates * p-value < 0.05; *** p-value < 0.001, two-way ANOVA followed by uncorrected Fisher's LSD, each comparison stands alone (**B**); Mann Whitney test (**C**).

**Appendix Figure S5.** ***Nirmatrelvir treatment does not impair the development of adaptive immune responses to VSV or LCMV.***

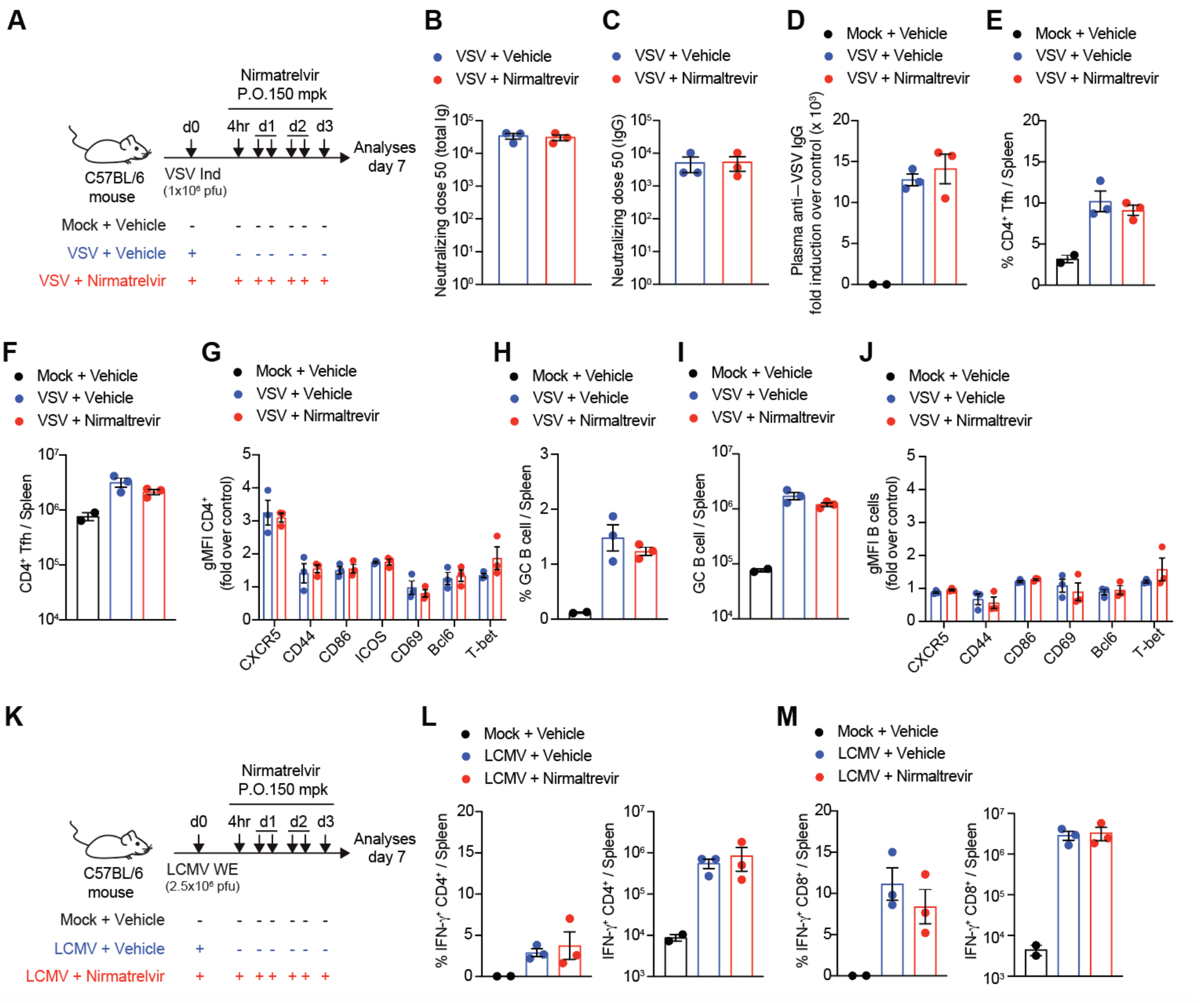


**(A)** Schematic representation of the experimental set up. C57BL/6 mice were intravenously infected with 1 x $10^6$ pfu of VSV Indiana.

**(B, C)** Neutralization dose 50 (ND50) of total immunoglobulin (Ig) (**B**) or IgG (**C**) against VSV in the plasma of the indicated mice.

**(D)** Quantification of anti-VSV IgG levels by ELISA in the plasma of the indicated mice.

**(E, F)** Frequency (**E**) and absolute number (**F**) of $CD4^+$ T follicular helper cells (Tfh) in the spleen of indicated mice. Tfh cells were defined as $live^+$/ $B220^-$/ $CD19^-$/ $CD8^-$/ $CD4^+$/ $CXCR5^+$/ $Bcl6^+$ cells.

**(G)** Geometric mean fluorescent intensity (gMFI) of markers expressed by $CD4^+$ T cells. Values are showed as fold expression over control.

**(H, I)** Frequency (**H**) and absolute number (**I**) of germinal center (GC) B cells in the spleen of indicated mice. GC B cells were defined as $live^+$/ $CD8^-$/ $CD4^-$/ $B220^+$/ $CD19^+$/ $GL7^+$ / $Bcl6^+$ cells.

**(J)** Geometric mean fluorescent intensity (gMFI) of markers expressed by B cells. Values are showed as fold expression over control.

**(K)** Schematic representation of the experimental set up. C57BL/6 mice were intravenously infected with $2.5 \times 10^6$ pfu of LCMV WE.

**(L, M)** Frequency (left panel) and absolute number (right panel) of IFN-$\gamma$–producing $CD4^+$ T cells **(L)** and $CD8^+$ T cells **(M)** in the spleen of indicated mice. Cells were stimulated *in vitro* with the LCMV peptides GP61 and GP33 for 4 hours at 37°C.

Data information: In **A** and **K**, infected mice were treated with 150 mpk of nirmatrelvir (red symbols, $n = 3$) or vehicle (blue symbols, $n = 3$) for six times by oral gavage (P.O.) starting 4 hours p.i., and every 12 hours thereafter. Mock-treated mice were used as non-infected controls (black symbols, $n = 2$). Blood and spleen were collected 7 days p.i.. Data are represented as mean ± SEM.

**Appendix Figure S6.** ***Effect of nirmatrelvir treatment on viral titers upon SARS-CoV-2 re-challenge.***

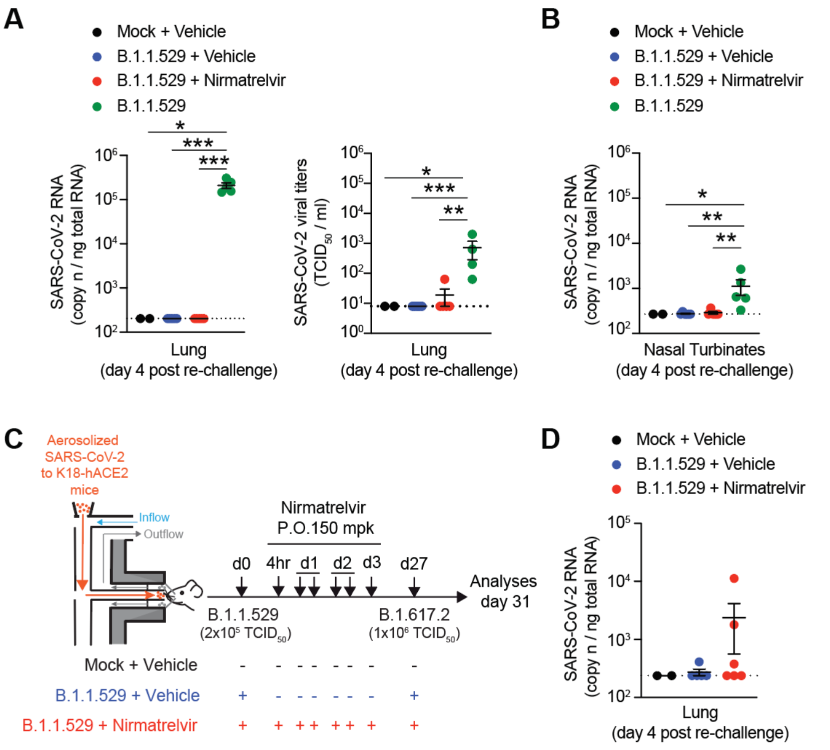


**(A)** Quantification of SARS-CoV-2 RNA (left panel) and viral titers (right panel) in the lungs of the indicated mice treated as described in **Figure 2A**. RNA values are expressed as copy number per ng of total RNA. Viral titers were determined by median tissue culture infectious dose ($TCID_{50}$). Limit of detection is indicated as a dotted line. Blue, vehicle-treated mice; red, nirmatrelvir-treated mice. Mock-treated mice were used as non-infected controls (black symbols). A group of naïve mice challenged with 1 x $10^6$ $TCID_{50}$ of SARS-CoV-2 B.1.1.529 served as additional controls (green symbols).

**(B)** Quantification of SARS-CoV-2 RNA in the nasal turbinates of the indicated mice 4 days post re-challenge. RNA values are expressed as copy number per ng of total RNA and the limit of detection is indicated as a dotted line.

**(C)** Schematic representation of the experimental set up. Non-anesthetized K18-hACE2 mice were exposed to a target dose of 2 x $10^5$ $TCID_{50}$ of aerosolized SARS-CoV-2 (B.1.1.529). Infected mice were treated with 150 mpk of nirmatrelvir (red symbols, *n* = 6) or vehicle (blue symbols, *n* = 6) for six times by oral gavage (P.O.) starting 4 hours p.i., and every 12 hours thereafter. Twenty-seven days after infection, mice were re-challenged with a target dose of 1 x $10^6$ $TCID_{50}$ of SARS-CoV-2 (B.1.617.2) through aerosol exposure. Mock-treated mice were used as non-infected controls (black symbols, *n* = 2).

**(D)** Quantification of SARS-CoV-2 RNA in the lung 4 days after re-challenge. RNA values are expressed as copy numbers per ng of total RNA and the limit of detection is indicated as a dotted line.

Data information: Data are represented as mean ± SEM and are representative of at least two independent experiments. * p-value < 0.05, ** p-value < 0.01, *** p-value < 0.001; Kruskal-Wallis test followed by uncorrect Dunn’s test, each comparison stands alone.

## Appendix Figure S7

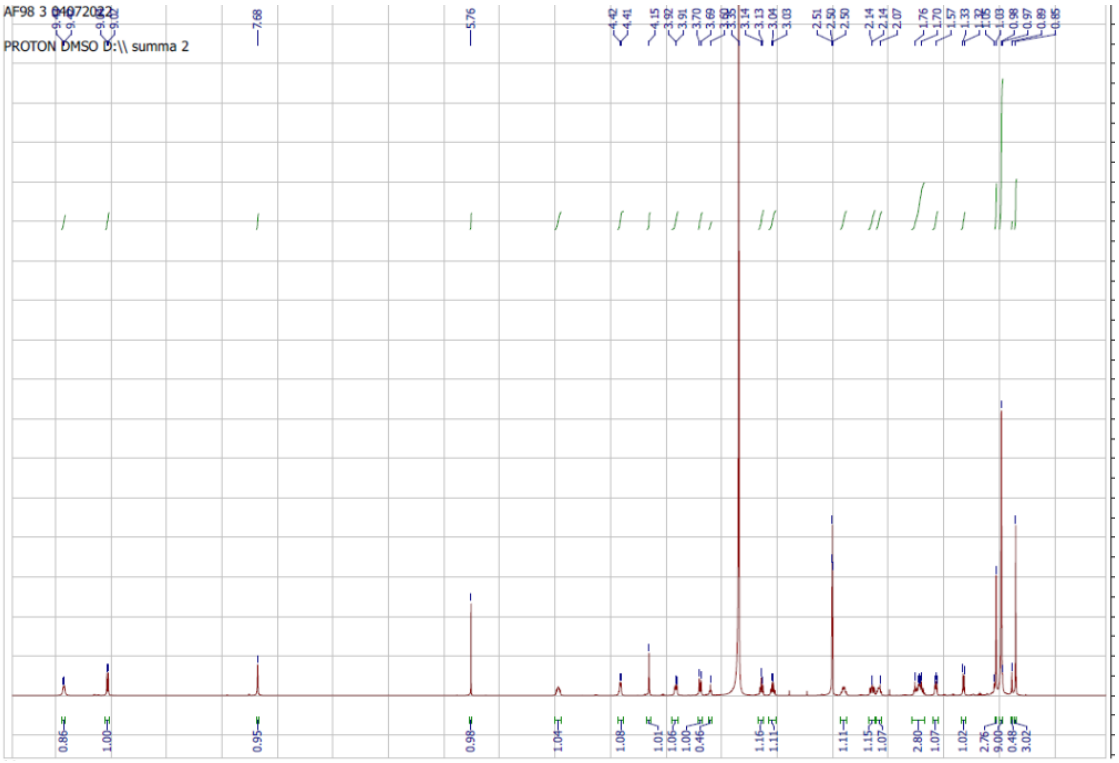


$^{1}$H NMR of Nirmatrelvir in DMSO-$d_6$ at 25°C

## Appendix Figure S8

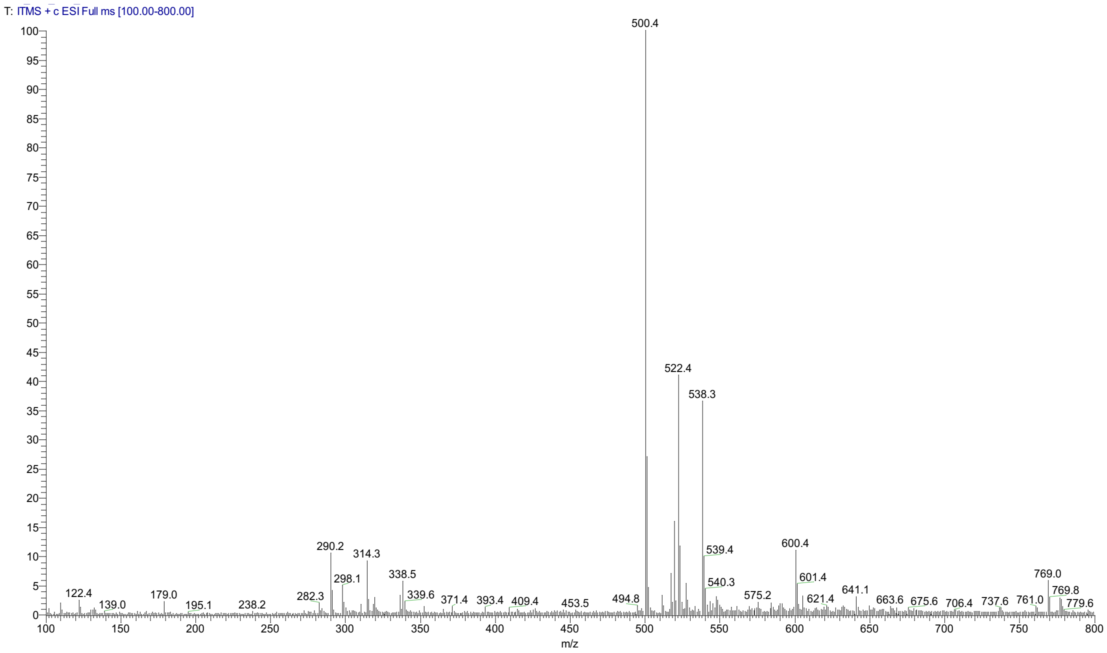


Mass spectra of Nirmatrelvir. MS (ESI) *m/z* calcd: $[M + H]^+$ for $C_{23}H_{33}F_3N_5O_4^+$ 500.5, found $[M+H]^+$ 500.4, $[M+Na]^+$ 522.4, $[M+K]^+$ 538.3.

**Appendix Table S1**

| | Q1 | Q3 | CE (V) | CXP (V) |
|---|---|---|---|---|
| Nirmatrelvir | 500.2 | 110.0 | 40 | 16 |
| | | 68.0 | 80 | 8 |
| JWH-250 (IS) | 336.1 | 121.0 | 31 | 18 |

LC-MS/MS Analysis. The parameters used for each analyte are listed in table.

**Appendix Table S2**

| Name | Clone | Source and catalog number | Dilution |
|---|---|---|---|
| CD8 | 53-6.7 | BD Biosciences #558106,<br>Biolegend #100759 | 1:100 |
| CD4 | RM4-5 | Biolegend #100548;<br>BD Biosciences #740208 | 1:100 |
| B220 | RA3-6B2 | BD Biosciences #564662 | 1:100 |
| CD19 | 1D3 | BD Biosciences #749027 | 1:100 |
| CD44 | IM7 | BD Biosciences #741227;<br>BioLegend #103028 | 1:150 |
| CD69 | H1.2F3 | BD Biosciences #612793 | 1:100 |
| CD19 | 1D3 | BD Biosciences #749027 | 1:100 |
| CD62L | MEL-4 | Biolegend #104453 | 1:100 |
| CD45 | 30-F11 | Biolegend #103113,<br>BD Biosciences #564279 | 1:100 |
| IFN-γ | XMG1.2 | BD Biosciences #557735 | 1:100 |
| TNF-α | MP6-XT22 | Biolegend #506329 | 1:100 |
| CD279 (PD-1) | RMP1-30 | BD Biosciences #749306 | 1:100 |
| Granzyme-B | GB12 | Invitrogen #MHGB04 | 1:80 |
| CD86 | GL1 | BD Biosciences #564199 | 1:100 |
| CD80 | 16-10A1 | Biolegend #104738 | 1:100 |
| CD95 | Jo2 | BD Biosciences #557653 | 1:100 |
| CD127 | SB/199 | BD Biosciences #560733 | 1:100 |
| KLRG1 | 2F1/KLRG1 | Biolegend #138409 | 1:100 |
| GL7 | GL7 | Biolegend # 144612 | 1:100 |
| Bcl6 | K112-91 | BD Biosciences #562401 | 1:70 |
| CXCR5 | 2G8 | Biolegend #145532 | 1:70 |
| T-bet | 4B10 | Invitrogen #25-5825-80 | 1:70 |
| ICOS | C398.4A | Biolegend #313537 | 1:100 |
| Streptavidin-AF647 | | Invitrogen # S32357 | |
| Streptavidin-AF488 | | Invitrogen #S32354 | |

List of antibodies (Abs) used for flow cytometry analysis.
