# Supplementary material for: Nirmatrelvir treatment of SARS‐CoV‐2‐infected mice blunts antiviral adaptive immune responses
Source: EMBO Mol Med. 2023 Mar 22;15(5):e17580. doi: 10.15252/emmm.202317580 (PMC10165354; doi:10.15252/emmm.202317580)
Supplement: Supplementary file 5 [file EMMM-15-e17580-s005.pdf]

EMBO Molecular Medicine

Nirmatrelvir treatment of SARS-CoV-2 infected mice blunts antiviral adaptive immune responses

Valeria Fumagalli, Pietro Di Lucia, Micol Ravà, Davide Marotta, Elisa Bono, Stefano Grassi, Lorena Donnici, Rolando Cannalire, Irina Stefanelli, Anastasia Ferraro, Francesca Esposito, Elena Pariani, Donato Inverso, Camilla Montesano, Serena Delbue, Stanley Perlman, Enzo Tramontano, Raffaele De Francesco, Vincenzo Summa, Luca Guidotti, and Matteo Iannacone
DOI: 10.15252/emmm.202317580

Corresponding author(s): Matteo Iannacone (iannacone.matteo@hsr.it) , Luca Guidotti (guidotti.luca@hsr.it)

Review Timeline:

Transfer from Review Commons:	15th Feb 23
Editorial Decision:	24th Feb 23
Revision Received:	27th Feb 23
Accepted:	2nd Mar 23

Review COMMONS

Editor: Zeljko Durdevic

Transaction Report: This manuscript was transferred to EMBO Molecular Medicine following peer review at Review Commons:

(Note: With the exception of the correction of typographical or spelling errors that could be a source of ambiguity, letters and reports are not edited. Depending on transfer agreements, referee reports obtained elsewhere may or may not be included in this compilation. Referee reports are anonymous unless the Referee chooses to sign their reports.)

Review #1

1. Evidence, reproducibility and clarity:

Evidence, reproducibility and clarity (Required)

In this study, Fumagalli et. al evaluated the impact of Nirmatrelvir drug treatment on the development of SARS-CoV-2-specific adaptive immune responses in a mice model. Nirmatrelvir is one of the component of Paxlovid drug that has been shown to reduce the risk of progression to severe COVID-19 and long COVID. Herein, authors show that nirmatrelvir administration early after infection blunts the development of SARS-CoV-2-specific antibody and T cell responses. Upon secondary challenge, nirmatrelvir-treated mice developed fewer memory T and B cells to the infected lungs and to mediastinal lymph nodes, respectively. Overall, the experimental methods, figures, results, statistical analysis and findings of this study are interesting and convincing.

Referees cross-commenting

I agree with the Reviewer 2 comments.

2. Significance:

Significance (Required)

It was known that nirmatrelvir reduces the risk of severe covid and long covid but, whether its treatment has any impact on adaptive immune response was not known/evaluated. This study has importantly addressed that impact of nirmatrelvir treatment can impair both T and B cell adaptive immune responses. It would have been impactful to understand the mechanism of T and B cell immune response impairment following nirmatrelvir treatment in mice which they have already mentioned a limitation of the study.

Moreover this study provides important implications for clinical management of COVID patients and to revise the treatment strategies to avoid virological and/or symptomatic relapse after Paxlovid/nirmatrelvir treatment completion that have been reported in some individuals.

I am not a mice model expert. Not sure whether the viral dose given to mice in this study was optimal to study the impact of the said drug.

3. How much time do you estimate the authors will need to complete the suggested revisions:

Estimated time to Complete Revisions (Required)

(Decision Recommendation)

Less than 1 month

4. *Review Commons* values the work of reviewers and encourages them to get credit for their work. Select 'Yes' below to register your reviewing activity at [Web of Science Reviewer Recognition Service]() (formerly Publons); note that the content of your review will not be visible on Web of Science.

Web of Science Reviewer Recognition

Yes

Review #2

1. Evidence, reproducibility and clarity:

Evidence, reproducibility and clarity (Required)

Summary:

In this manuscript, the authors show that Paxlovid, a commonly used antiviral for SARS-CoV-2 infections, blunts the adaptive immune response to the virus. Indeed, they show convincing effects on T cell and B cell responses in the K18-hACE2 mouse model infected with Omicron variant. The effect is observed when drug treatment was started at 4, 24, or 48 h post infection. Experiments are well done and the data are presented clearly. However, the early timing of drug administration resulted in minimal virus replication, thus likely limiting innate immune activation and antigenic exposure. Indeed, the authors show that the drug did not decrease adaptive responses to other viral infections, indicating that the effect on adaptive immunity in SARS-CoV-2 infection can be explained by decreased viral antigen production. Whether this is the mechanism by which relapse infections occur in humans after Paxlovid treatment is unclear.

Major comments:

The authors should discuss whether the timing of drug administration in their experiments is relevant to the timing of when Paxlovid is commonly started in humans.

Does Paxlovid limit the adaptive immune response when given later in infection?

Omicron variant has limited replication in the K18 mouse model and does not cause disease. Thus, the authors are starting from a model with artificially limited viral antigen production.

Does Paxlovid limit the adaptive immune response when given during an infection with a variant strain that replicates robustly in the K18 mice?

2. Significance:

Significance (Required)

Nirmatrelvir/Paxlovid is used clinically for treatment of COVID-19. Relapse infections have been reported after courses of the drug. The authors show here that Paxlovid treatment during a mouse model of SARS-CoV-2 infection results in diminished induction of adaptive immunity and immune memory. This is most probably due to decreased production of viral antigenic stimuli due to inhibition of virus replication. The concept that less viral antigen will result in less induction of immunity is not surprising. Further, whether the phenomenon observed here in a mouse model with poor susceptibility to the chosen virus strain is related to relapse infections in humans was not established. Nonetheless, the audience for this work is broad and this work could be of interest due to the common use of Paxlovid and the ongoing SARS-CoV-2 infections across the world.

3. How much time do you estimate the authors will need to complete the suggested revisions:

Estimated time to Complete Revisions (Required)

(Decision Recommendation)

Between 1 and 3 months

4. *Review Commons* values the work of reviewers and encourages them to get credit for their work. Select 'Yes' below to register your reviewing activity at [Web of Science Reviewer Recognition Service] (formerly Publons); note that the content of your review will not be visible on Web of Science.

Web of Science Reviewer Recognition

No

Response to Reviewers' Comments on Fumagalli et al. "Nirmatrelvir treatment blunts the development of antiviral adaptive immune responses in SARS-CoV-2 infected mice" (Preprint RC-2022-01777).

We wish to thank the reviewers for the scholarly review of our work and the very helpful comments. Based on their constructive suggestions, we have generated substantial new experimental data that, in our opinion, positively address all the major and minor concerns raised. In particular, we have confirmed the negative impact of nirmatrelvir treatment on adaptive immune responses in setting of robust SARS-CoV-2 replication (Delta infection in K18-hACE2 transgenic mice and mouse-adapted SARS-CoV-2 infection of wild-type mice). One main and one supplemental figure have been added in response to the reviewers' comments. One additional figure – termed Reviewer Figure 1 – has been included in this letter for the reviewers' benefit; while addressing specific comments, we believe that the data depicted in this latter figure remain tangential to the main message of our work and, as such, it should not be incorporated in the final version. To aid the reviewers in the re-evaluation of this study, all relevant passages in the revised text have been written in red. A summary of the changes made to the figures and tables is provided as an appendix at the end of this letter.

Reviewers' comments:

Reviewer #1 (Evidence, reproducibility and clarity (Required)):

In this study, Fumagalli et. al evaluated the impact of Nirmatrelvir drug treatment on the development of SARS-CoV-2-specific adaptive immune responses in a mice model. Nirmatrelvir is one of the component of Paxlovid drug that has been shown to reduce the risk of progression to severe COVID-19 and long COVID. Herein, authors show that nirmatrelvir administration early after infection blunts the development of SARS-CoV-2-specific antibody and T cell responses. Upon secondary challenge, nirmatrelvir-treated mice developed fewer memory T and B cells to the infected lungs and to mediastinal lymph nodes, respectively. Overall, the experimental methods, figures, results, statistical analysis and findings of this study are interesting and convincing.

We wish to thank the reviewer for the overall positive assessment of our work.

CROSS-CONSULATION COMMENTS

I agree with the Reviewer 2 comments.

Reviewer #1 (Significance (Required))**:**

It was known that nirmatrelvir reduces the risk of severe covid and long covid but, whether its treatment has any impact on adaptive immune response was not known/evaluated. This study has importantly addressed that impact of nirmatrelvir treatment can impair both T and B cell adaptive immune responses. It would have been impactful to understand the mechanism of T and B cell immune response impairment following nirmatrelvir treatment in mice which they have already mentioned a limitation of the study.

We agree with this reviewer that the mechanism of T and B cell impairment following nirmatrelvir treatment should be addressed in future studies.

Moreover this study provides important implications for clinical management of COVID patients and to revise the treatment strategies to avoid virological and/or symptomatic relapse after Paxlovid/nirmatrelvir treatment completion that have been reported in some individuals.

We thank the reviewer for highlighting the impact of our results.

I am not a mice model expert. Not sure whether the viral dose given to mice in this study was optimal to study the impact of the said drug.

Depending on the virus used, we infected mice with 10^5-10^6 $TCID_{50}$. This is in line with most studies of SARS-CoV-2 infection in mice. It is difficult to know what the average infectious dose in humans is, but the human challenge trial in young adults shows that exposure of individuals to as low as 10 $TCID_{50}$ of SARS-CoV-2 led to detectable viral RNA in the upper airways (Killingley *et al*, 2022).

Reviewer #2 (Evidence, reproducibility and clarity (Required)):

Summary:

In this manuscript, the authors show that Paxlovid, a commonly used antiviral for SARS-CoV-2 infections, blunts the adaptive immune response to the virus. Indeed, they show convincing effects on T cell and B cell responses in the K18-hACE2 mouse model infected with Omicron variant. The effect is observed when drug treatment was started at 4, 24, or 48 h post infection. Experiments are well done and the data are presented clearly.

We thank the reviewer for the overall positive assessment of our work.

However, the early timing of drug administration resulted in minimal virus replication, thus likely limiting innate immune activation and antigenic exposure. Indeed, the authors show that the drug did not decrease adaptive responses to other viral infections, indicating that the effect on adaptive immunity in SARS-CoV-2 infection can be explained by decreased viral antigen production. Whether this is the mechanism by which relapse infections occur in humans after Paxlovid treatment is unclear.

Major comments:

The authors should discuss whether the timing of drug administration in their experiments is relevant to the timing of when Paxlovid is commonly started in humans.
Does Paxlovid limit the adaptive immune response when given later in infection?

We thank the reviewer for raising this valid comment. First, we would like to point out that the kinetics of viral replication upon SARS-CoV-2 exposure differ between mice and humans. When mice are exposed to a high-dose (10^5 $TCID_{50}$) aerosolized SARS-CoV-2, they show peak viral replication in the airways at day 3 post exposure and viral RNA is undetectable in the upper and lower airways after day 7 (**Reviewer Figure 1A** and (Fumagalli *et al,* 2021)). It is difficult to extract precise data in humans, but the human challenge trial in young adults shows that exposure of individuals to an extremely low dose (10 $TCID_{50}$!) of SARS-CoV-2 led to the detection of viral RNA in the upper airways for longer than 14 days (**Reviewer Figure 1B** and (Killingley *et al,* 2022)). Therefore, it is very difficult to estimate what would possibly mimic what is occurring in treated COVID-19 patients, especially in line of the current COVID-19 guidelines for ritonavir-boosted nirmatrelvir that suggests to initiate treatment as soon as possible and within 5 days of symptoms (https://www.covid19treatmentguidelines.nih.gov/therapies/antiviral-therapy/ritonavir-boosted-nirmatrelvir--paxlovid-/). The choice to start treatment 4 hours after infection was

motivated by the original paper that reported *in vivo* antiviral activity of nirmatrelvir against SARS-CoV-2 (Owen *et al*, 2021). That said, we performed additional experiments whereby we treated mice with nirmatrelvir 24 or 48 hours after infection (at or near the peak of viral replication). As shown in the new **Figure 3**, such treatment also resulted in blunted adaptive immune responses.

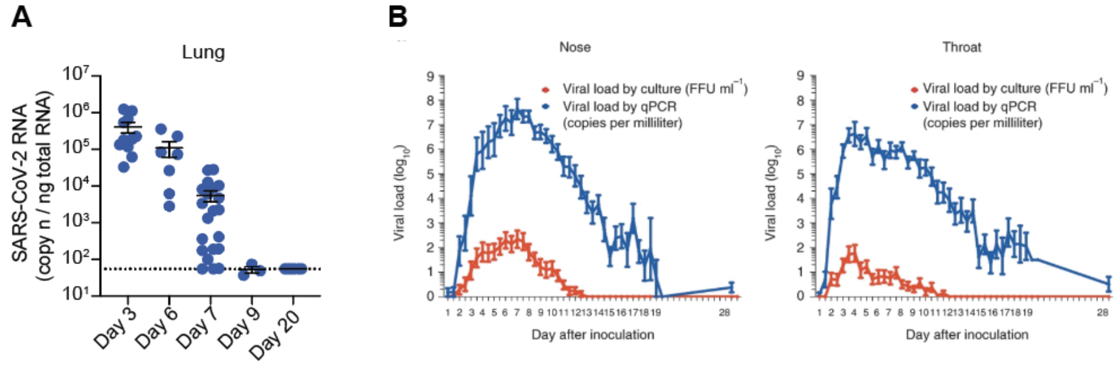

***Reviewer Figure 1**. **(A)** K18-hACE2 mice were exposed to a target dose of 2 x 10^5 $TCID_{50}$ of aerosolized SARS-CoV-2 (D614G). Quantification of SARS-CoV-2 RNA in the lung after infection. RNA values are expressed as copy numbers per ng of total RNA and the limit of detection is indicated as a dotted line. **(B)** Healthy adult volunteers were challenged intranasally with SARS-CoV-2. In the infected individuals (n = 18 biologically independent participants). Viral load in twice-daily nose and throat swab samples was measured by qPCR (blue) and focus-forming assay (red) (a). Results are expressed as mean ± SEM. Adapted from ref.* (Killingley *et al*, 2022).

Omicron variant has limited replication in the K18 mouse model and does not cause disease. Thus, the authors are starting from a model with artificially limited viral antigen production. Does Paxlovid limit the adaptive immune response when given during an infection with a variant strain that replicates robustly in the K18 mice?

We thank the reviewer for raising this issue. In the revised manuscript we have now performed experiments where we infected K18-hACE2 transgenic mice with the Delta (B.1.617.2) variant, known to replicate at higher level compared to the Omicron variants (Shuai *et al*, 2022). Additionally, we have infected WT mice with a mouse-adapted SARS-CoV-2 (rSARS2-N501Y_{MA30})(Wong *et al*, 2022) that replicates robustly and induces significant disease. These new results, now shown in the new **Figure 3** and new **Figure S4**, confirm that nirmatrelvir treatment blunts the development of antiviral adaptive immune responses regardless of the variants/strain used for infection.

Reviewer #2 (Significance (Required)):

Significance:

Nirmatrelvir/Paxlovid is used clinically for treatment of COVID-19. Relapse infections have been reported after courses of the drug. The authors show here that Paxlovid treatment during a mouse model of SARS-CoV-2 infection results in diminished induction of adaptive immunity and immune memory. This is most probably due to decreased production of viral antigenic stimuli due to inhibition of virus replication. The concept that less viral antigen will result in less induction of immunity is not surprising. Further, whether the phenomenon observed here in a mouse model with poor susceptibility to the chosen virus strain is related to relapse infections in humans was not established. Nonetheless, the audience for this work is broad and this work could be of interest due to the common use of Paxlovid and the ongoing SARS-CoV-2 infections across the world.

Although we did not investigate the mechanism underlying the reported observation in depth, we agree with this reviewer that the most likely explanation for the reduced adaptive immune responses is decreased production of viral antigens. In this regard, it is probably not terribly surprising. However, it is worth noting that successful antimicrobial treatment does not inevitably result in reduced adaptive immune responses to any pathogen. For instance, treatment of mice infected with *Listeria monocytogenes* with amoxicillin early after infection did not significantly impair the development of T cell responses (Corbin & Harty, 2004; Mercado *et al*, 2000). Furthermore, treatment with antibiotics before *L. monocytogenes* infection allowed the development of functional antigen-specific memory $CD8^+$ T cells in the absence of contraction (Badovinac *et al*, 2004). An additional, and possibly more relevant, example was published during the revision process: monoclonal antibody therapy with bamlanivimab during acute COVID-19 did not impact the development of a robust antiviral T cell response (Ramirez *et al*, 2022).

As per the comment related the poor susceptibility of the mouse model to the Omicron variants of SARS-CoV-2, we believe that the new data obtained with the Delta variant and with the mouse-adapted SARS-CoV-2 (new **Figure 3** and **S4**) convincingly show that nirmatrelvir treatment blunts antiviral adaptive immune responses to SARS-CoV-2 in mice.

List of modifications

New figures:

- **Figure 3**: new data as per reviewer’s suggestion.
- **Figure S4**: new data as per reviewer’s suggestion.

References

Badovinac VP, Porter BB & Harty JT (2004) CD8+ T cell contraction is controlled by early inflammation. *Nat Immunol* 5: 809–817

Corbin GA & Harty JT (2004) Duration of Infection and Antigen Display Have Minimal Influence on the Kinetics of the CD4+ T Cell Response to Listeria monocytogenes Infection. *J Immunol* 173: 5679–5687

Fumagalli V, Ravà M, Marotta D, Lucia PD, Laura C, Sala E, Grillo M, Bono E, Giustini L, Perucchini C, *et al* (2021) Administration of aerosolized SARS-CoV-2 to K18-hACE2 mice uncouples respiratory infection from fatal neuroinvasion. *Sci Immunol* 7: eabl9929

Killingley B, Mann AJ, Kalinova M, Boyers A, Goonawardane N, Zhou J, Lindsell K, Hare SS, Brown J, Frise R, *et al* (2022) Safety, tolerability and viral kinetics during SARS-CoV-2 human challenge in young adults. *Nat Med* 28: 1031–1041

Mercado R, Vijh S, Allen SE, Kerksiek K, Pilip IM & Pamer EG (2000) Early Programming of T Cell Populations Responding to Bacterial Infection. *J Immunol* 165: 6833–6839

Owen DR, Allerton CMN, Anderson AS, Aschenbrenner L, Avery M, Berritt S, Boras B, Cardin RD, Carlo A, Coffman KJ, *et al* (2021) An oral SARS-CoV-2 Mpro inhibitor clinical candidate for the treatment of COVID-19. *Science* 374: 1586–1593

Ramirez SI, Grifoni A, Weiskopf D, Parikh UM, Heaps A, Faraji F, Sieg SF, Ritz J, Moser C, Eron JJ, *et al* (2022) Bamlanivimab therapy for acute COVID-19 does not blunt SARS-CoV-2-specific memory T cell responses. *Jci Insight* 7

Shuai H, Chan JF-W, Hu B, Chai Y, Yuen TT-T, Yin F, Huang X, Yoon C, Hu J-C, Liu H, *et al* (2022) Attenuated replication and pathogenicity of SARS-CoV-2 B.1.1.529 Omicron. *Nature* 603: 693–699

Wong L-YR, Zheng J, Wilhelmsen K, Li K, Ortiz ME, Schnicker NJ, Thurman A, Pezzulo AA, Szachowicz PJ, Li P, *et al* (2022) Eicosanoid signaling blockade protects middle-aged mice from severe COVID-19. *Nature*: 1–9

24th Feb 2023

Dear Prof. Iannacone,

Thank you for the submission of your revised manuscript to EMBO Molecular Medicine. I am pleased to inform you that we will be able to accept your manuscript pending the following final amendments:

1) Manuscript type: Please format your manuscript as a report article. Please check "Author Guidelines" for more information.
https://www.embopress.org/page/journal/17574684/authorguide#reportsarticleguide
2) Figures: Please upload separate, high-resolution main figure files. Compile all supplementary figures with their legends into an Appendix file with a table of content on the first page. Rename the figures to 'Appendix Figure S1" etc. also in the main text. Please move images of Nirmatrelvir 1H NMR and MS spectra form M&M figures to an Appendix figure and add callouts at the appropriate place in M&M. Please check "Author Guidelines" for more information:
https://www.embopress.org/page/journal/17574684/authorguide#figureformat
3) Tables: Move both tables from M&M section to Appendix. Tables should be labeled 'Appendix Table S1' etc., with a title and short description. Please add callouts at the appropriate place in M&M.
4) In the main manuscript file, please do the following:
- Correct/answer the track changes suggested by our data editors by working from the attached document.
- Add callouts for Figure 2A and correct the callout for Figure 4S to S4.
- In M&M, provide the antibody dilutions that were used for each antibody.
- Please rename "Competing Interest Statement" to "Disclosure Statement & Competing Interests". We updated our journal's competing interests policy in January 2022 and request authors to consider both actual and perceived competing interests. Please review the policy https://www.embopress.org/competing-interests and update your competing interests if necessary.
- Author contributions: Please remove it from the manuscript and specify author contributions in our submission system. CRediT has replaced the traditional author contributions section because it offers a systematic machine-readable author contributions format that allows for more effective research assessment. You are encouraged to use the free text boxes beneath each contributing author's name to add specific details on the author's contribution. More information is available in our guide to authors:
https://www.embopress.org/page/journal/17574684/authorguide#authorshipguidelines
- In data availability, if no data are deposited in public repositories, please add the sentence: "This study includes no data deposited in external repositories".
Please check "Author Guidelines" for more information.
https://www.embopress.org/page/journal/17574684/authorguide#availabilityofpublishedmaterial
5) Funding: Please make sure the information about funding are complete in both our submission systema and in the manuscript.
6) Synopsis: Every published paper now includes a 'Synopsis' to further enhance discoverability. Synopses are displayed on the journal webpage and are freely accessible to all readers. They include separate synopsis image and synopsis text.
- Synopsis image: Please provide the visual abstract as a high-resolution jpeg file 550 px-wide x (250-400)-px high.
- Synopsis text: Please provide a short standfirst (maximum of 300 characters, including space) as well as 2-5 one sentence bullet points that summarise the paper as a .doc file. Please write the bullet points to summarise the key NEW findings. They should be designed to be complementary to the abstract - i.e. not repeat the same text. We encourage inclusion of key acronyms and quantitative information (maximum of 30 words / bullet point). Please use the passive voice.
- Please check your synopsis text and image before submission with your revised manuscript. Please be aware that in the proof stage minor corrections only are allowed (e.g., typos).
7) For more information: This space should be used to list relevant web links for further consultation by our readers. Could you identify some relevant ones and provide such information as well? Some examples are patient associations, relevant databases, OMIM/proteins/genes links, author's websites, etc...
8) As part of the EMBO Publications transparent editorial process initiative (see our Editorial at http://embomolmed.embopress.org/content/2/9/329), EMBO Molecular Medicine will publish online a Review Process File (RPF) to accompany accepted manuscripts. This file will be published in conjunction with your paper and will include the anonymous referee reports, your point-by-point response and all pertinent correspondence relating to the manuscript. Let us know whether you agree with the publication of the RPF and as here, if you want to remove or not any figures from it prior to publication. Please note that the Authors checklist will be published at the end of the RPF.
9) Please provide a point-by-point letter INCLUDING my comments as well as the reviewer's reports and your detailed responses (as Word file).

I look forward to reading a new revised version of your manuscript as soon as possible.

Yours sincerely,

Zeljko Durdevic

***** Reviewer's comments *****

Referee #1 (Remarks for Author):

The authors have adequately addressed my previous concerns. Particularly, the new experiments with the SARS-CoV-2 delta strain and mouse adapted strain are critical additions that support the overall conclusions of the manuscript.

The authors performed the requested editorial changes.

We are pleased to inform you that your manuscript is accepted for publication and is now being sent to our publisher to be included in the next available issue of EMBO Molecular Medicine.

EMBO Press Author Checklist

Corresponding Author Name: Matteo Iannacone	
Journal Submitted to: EMBO Molecular Medicine	
Manuscript Number: EMM-2023-17580	

USEFUL LINKS FOR COMPLETING THIS FORM
[The EMBO Journal - Author Guidelines]
[EMBO Reports - Author Guidelines]
[Molecular Systems Biology - Author Guidelines]
[EMBO Molecular Medicine - Author Guidelines]

Reporting Checklist for Life Science Articles (updated January 2022)
This checklist is adapted from Materials Design Analysis Reporting (MDAR) Checklist for Authors. MDAR establishes a minimum set of requirements in transparent reporting in the life sciences (see Statement of Task: 10.31222/osf.io/9sm4x). Please follow the journal's guidelines in preparing your manuscript.
Please note that a copy of this checklist will be published alongside your article.

Abridged guidelines for figures
1. Data
The data shown in figures should satisfy the following conditions:
- the data were obtained and processed according to the field's best practice and are presented to reflect the results of the experiments in an accurate and unbiased manner.
- ideally, figure panels should include only measurements that are directly comparable to each other and obtained with the same assay.
- plots include clearly labeled error bars for independent experiments and sample sizes. Unless justified, error bars should not be shown for technical replicates.
- if n<5, the individual data points from each experiment should be plotted. Any statistical test employed should be justified.
- Source Data should be included to report the data underlying figures according to the guidelines set out in the authorship guidelines on Data Presentation.

2. Captions
Each figure caption should contain the following information, for each panel where they are relevant:
- a specification of the experimental system investigated (eg cell line, species name).
- the assay(s) and method(s) used to carry out the reported observations and measurements.
- an explicit mention of the biological and chemical entity(ies) that are being measured.
- an explicit mention of the biological and chemical entity(ies) that are altered/varied/perturbed in a controlled manner.
- the exact sample size (n) for each experimental group/condition, given as a number, not a range;
- a description of the sample collection allowing the reader to understand whether the samples represent technical or biological replicates (including how many animals, litters, cultures, etc.).
- a statement of how many times the experiment shown was independently replicated in the laboratory.
- definitions of statistical methods and measures:

  - common tests, such as t-test (please specify whether paired vs. unpaired), simple χ^2 tests, Wilcoxon and Mann-Whitney tests, can be unambiguously identified by name only, but more complex techniques should be described in the methods section;
  - are tests one-sided or two-sided?
  - are there adjustments for multiple comparisons?
  - exact statistical test results, e.g., P values = x but not P values < x;
  - definition of 'center values' as median or average;
  - definition of error bars as s.d. or s.e.m.

Please complete ALL of the questions below.
Select "Not Applicable" only when the requested information is not relevant for your study.

Materials

Newly Created Materials	Information included in the manuscript?	In which section is the information available? (Reagents and Tools Table, Materials and Methods, Figures, Data Availability Section)
New materials and reagents need to be available; do any restrictions apply?	Yes	Materials and Methods

Antibodies	Information included in the manuscript?	In which section is the information available? (Reagents and Tools Table, Materials and Methods, Figures, Data Availability Section)
For **antibodies** provide the following information:<br>- Commercial antibodies: RRID (if possible) or supplier name, catalogue number and or/clone number<br>- Non-commercial: RRID or citation	Yes	Materila and Methods and Appendix file

DNA and RNA sequences	Information included in the manuscript?	In which section is the information available? (Reagents and Tools Table, Materials and Methods, Figures, Data Availability Section)
**Short novel DNA or RNA including primers, probes**: provide the sequences.	Not Applicable	

Cell materials	Information included in the manuscript?	In which section is the information available? (Reagents and Tools Table, Materials and Methods, Figures, Data Availability Section)
**Cell lines:** Provide species information, strain. Provide accession number in repository **OR** supplier name, catalog number, clone number, and/**OR** RRID.	Not Applicable	
**Primary cultures:** Provide species, strain, sex of origin, genetic modification status.	Not Applicable	
Report if the cell lines were recently **authenticated** (e.g., by STR profiling) and tested for mycoplasma contamination.	Not Applicable	

Experimental animals	Information included in the manuscript?	In which section is the information available? (Reagents and Tools Table, Materials and Methods, Figures, Data Availability Section)
**Laboratory animals or Model organisms:** Provide species, strain, sex, age, genetic modification status. Provide accession number in repository **OR** supplier name, catalog number, clone number, **OR** RRID.	Yes	Materials and Methods and Results
**Animal observed in or captured from the field:** Provide species, sex, and age where possible.	Not Applicable	
Please detail **housing and husbandry conditions**.	Yes	Materials and Methods

Plants and microbes	Information included in the manuscript?	In which section is the information available? (Reagents and Tools Table, Materials and Methods, Figures, Data Availability Section)
**Plants:** provide species and strain, ecotype and cultivar where relevant, unique accession number if available, and source (including location for collected wild specimens).	Not Applicable	
**Microbes:** provide species and strain, unique accession number if available, and source.	Yes	Materials and Methods

Human research participants	Information included in the manuscript?	In which section is the information available? (Reagents and Tools Table, Materials and Methods, Figures, Data Availability Section)
If collected and within the bounds of privacy constraints report on age, sex and gender or ethnicity for all study participants.	Not Applicable	

Core facilities	Information included in the manuscript?	In which section is the information available? (Reagents and Tools Table, Materials and Methods, Figures, Data Availability Section)
If your work benefited from core facilities, was their service mentioned in the acknowledgments section?	Yes	Acknowledgments

Design

Study protocol	Information included in the manuscript?	In which section is the information available? (Reagents and Tools Table, Materials and Methods, Figures, Data Availability Section)
If study protocol has been **pre-registered, provide DOI in the manuscript**. For clinical trials, provide the trial registration number **OR** cite DOI.	Not Applicable	
Report the **clinical trial registration number** (at ClinicalTrials.gov or equivalent), where applicable.	Not Applicable	

Laboratory protocol	Information included in the manuscript?	In which section is the information available? (Reagents and Tools Table, Materials and Methods, Figures, Data Availability Section)
Provide DOI OR other citation details if **external detailed step-by-step protocols** are available.	Not Applicable	

Experimental study design and statistics	Information included in the manuscript?	In which section is the information available? (Reagents and Tools Table, Materials and Methods, Figures, Data Availability Section)
Include a statement about **sample size** estimate even if no statistical methods were used.	Yes	Figures and Materials and Methods
Were any steps taken to minimize the effects of subjective bias when allocating animals/samples to treatment (e.g. **randomization procedure**)? If yes, have they been described?	Not Applicable	
Include a statement about **blinding** even if no blinding was done.	Not Applicable	
Describe **inclusion/exclusion criteria** if samples or animals were excluded from the analysis. Were the criteria pre-established?<br><br>If sample or data points were omitted from analysis, report if this was due to attrition or intentional exclusion and provide justification.	Not Applicable	
For every figure, are **statistical tests** justified as appropriate? Do the data meet the assumptions of the tests (e.g., normal distribution)? Describe any methods used to assess it. Is there an estimate of variation within each group of data? Is the variance similar between the groups that are being statistically compared?	Yes	Figures and Materials and Methods

Sample definition and in-laboratory replication	Information included in the manuscript?	In which section is the information available? (Reagents and Tools Table, Materials and Methods, Figures, Data Availability Section)
In the figure legends: state number of times the experiment was **replicated** in laboratory.	Yes	Figure legends
In the figure legends: define whether data describe **technical or biological replicates**.	Yes	Figure legends

Ethics

Ethics	Information included in the manuscript?	In which section is the information available? (Reagents and Tools Table, Materials and Methods, Figures, Data Availability Section)
Studies involving **human participants**: State details of **authority granting ethics approval** (IRB or equivalent committee(s), provide reference number for approval.	Not Applicable	
Studies involving **human participants**: Include a statement confirming that **informed consent** was obtained from all subjects and that the experiments conformed to the principles set out in the WMA Declaration of Helsinki and the Department of Health and Human Services Belmont Report.	Not Applicable	
Studies involving **human participants:** For publication of **patient photos**, include a statement confirming that consent to publish was obtained.	Not Applicable	
Studies involving experimental **animals**: State details of **authority granting ethics approval** (IRB or equivalent committee(s), provide reference number for approval. Include a statement of compliance with ethical regulations.	Yes	n° 270/2022-PR (prot. 6EEAF.228)
Studies involving **specimen and field samples:** State if relevant **permits** obtained, provide details of authority approving study; if none were required, explain why.	Not Applicable	

Dual Use Research of Concern (DURC)	Information included in the manuscript?	In which section is the information available? (Reagents and Tools Table, Materials and Methods, Figures, Data Availability Section)
Could your study fall under dual use research restrictions? Please check biosecurity documents and list of **select agents and toxins** (CDC): https://www.selectagents.gov/sat/list.htm	Not Applicable	
If you used a select agent, is the security level of the lab appropriate and reported in the manuscript?	Not Applicable	
If a study is subject to dual use research of concern regulations, is the name of the **authority granting approval and reference number** for the regulatory approval provided in the manuscript?	Not Applicable	

Reporting

The MDAR framework recommends adoption of discipline-specific guidelines, established and endorsed through community initiatives. Journals have their own policy about requiring specific guidelines and recommendations to complement MDAR.

Adherence to community standards	Information included in the manuscript?	In which section is the information available? (Reagents and Tools Table, Materials and Methods, Figures, Data Availability Section)
State if relevant guidelines or checklists (e.g., **ICMJE, MIBBI, ARRIVE, PRISMA**) have been followed or provided.	Not Applicable	
For **tumor marker prognostic studies**, we recommend that you follow the **REMARK** reporting guidelines (see link list at top right). See author guidelines, under 'Reporting Guidelines'. Please confirm you have followed these guidelines.	Not Applicable	
For **phase II and III randomized controlled trials**, please refer to the **CONSORT** flow diagram (see link list at top right) and submit the CONSORT checklist (see link list at top right) with your submission. See author guidelines, under 'Reporting Guidelines'. Please confirm you have submitted this list.	Not Applicable	

Data Availability

Data availability	Information included in the manuscript?	In which section is the information available? (Reagents and Tools Table, Materials and Methods, Figures, Data Availability Section)
Have **primary datasets** been deposited according to the journal's guidelines (see 'Data Deposition' section) and the respective accession numbers provided in the Data Availability Section?	Not Applicable	
Were **human clinical and genomic datasets** deposited in a public access-controlled repository in accordance to ethical obligations to the patients and to the applicable consent agreement?	Not Applicable	
Are **computational models** that are central and integral to a study available without restrictions in a machine-readable form? Were the relevant accession numbers or links provided?	Not Applicable	
If publicly available data were reused, provide the respective **data citations in the reference list**.	Not Applicable	
